# Antifilarial Lead Molecules Isolated from *Trachyspermum ammi*

**DOI:** 10.3390/molecules13092156

**Published:** 2008-09-11

**Authors:** Nisha Mathew, Shailja Misra-Bhattacharya, Vanamail Perumal, Kalyanasundaram Muthuswamy

**Affiliations:** 1 Vector Control Research Centre (ICMR), Indira Nagar, Pondicherry-605006, India. E-mails: vanamail@yahoo.com (V. P.); kannananthi@yahoo.com (K. M.); 2 Division of Parasitology, Central Drug Research Institute, Lucknow 226001, India. E-mail: shailjacdri@yahoo.com (S. M-B.)

**Keywords:** Antifilarial, Thymol, *Brugia malayi*, *Setaria digitata*, *Trachyspermum ammi*

## Abstract

Lymphatic filariasis is caused by infection with the parasitic filarial nematodes *Wuchereria bancrofti, Brugia malayi* and *B. timori*, transmitted by mosquitoes. The lack of an adulticidal drug poses a challenge to filariasis elimination, hence it is essential to develop an effective antifilarial drug which could either kill or permanently sterilize the adult worms. In the reported work the *in vitro* activity of a methanolic extract of fruits of *Trachyspermum ammi* (Apiaceae) against adult bovine filarial *Setaria digitata* worms has been investigated. A bioassay-guided fractionation was carried out by subjecting the crude extract to flash chromatography. HPLC analysis was done for the crude extract and active fraction. The crude extract and the active fraction showed significant activity against the adult *S. digitata* by both a worm motility and MTT [3-(4,5-dimethylthiazol-2-yl)-2,5-diphenyltetrazolium bromide] reduction assays. The isolated active principle was chemically characterized by IR, ^1^H-NMR and MS analysis and identified as a phenolic monoterpene. It was screened for *in vivo* antifilarial activity against the human filarial worm *B. malayi* in *Mastomys coucha*, showing macrofilaricidal activity and female worm sterility *in vivo* against *B. malayi*. The findings thus provide a new lead for development of a macrofilaricidal drug from natural products.

## Introduction

Lymphatic filariasis is caused by the infection with parasitic filarial nematodes *Wuchereria bancrofti, Brugia malayi* and *B. timori*, transmitted by mosquito vectors. The drugs currently used for lymphatic filariasis include annual doses of the microfilaricidal drug DEC (diethylcarbamazine), DEC plus albendazole, ivermectin plus albendazole or the use of DEC fortified salt [1]. None of these is effective in killing the adult worms, which can live in the host for several years [2] and the treatments are therefore aimed solely at reducing transmission and pathology. Despite the important addition to our knowledge of newer molecules [3,4,5,6] with antifilarial activity, none has developed fruitfully as a macrofilaricidal drug due to their low antifilarial activity and/or high toxicity. This warrants the need for developing an effective and safe drug to kill or permanently sterilize the adult worms. One of the methods for identifying leads for drug development is to screen medicinal plants for the required activity. *Trachyspermum ammi* (Ajwain caroway, family Apiaceae) is known for its antiviral [7], anti-inflammatory [8], antifungal [9,10,11,12,13], molluscicidal [14,15,16], antihelminthic (in sheep) [17], plant nematicidal [18], antipyretic [19], antiaggregatory [20] and antimicrobial activity [21,22]. This study reports the antifilarial potential of the fruit extract of *T. ammi* against the adult bovine filarial worm *Setaria digitata*. A compound obtained from *T. ammi* was identified and purified by bioassay-guided chemical fractionation and screened for antifilarial activity against *B*. *malayi* in an animal model.

## Results and Discussion

### Plant extraction, purification and characterization

The yields of the crude residue after removal of the solvent from the *T.ammi* extract and the active fraction after silica gel column chromatography (SGCC) were 14.15% w/w and 5.76% w/w respectively. The residue of the crude extract was brownish coloured oily liquid. On repeated chromatographic purification the active fraction yielded a white crystalline solid that by combined FT-IR, NMR and mass spectral analysis was identified as 2-isopropyl-5-methyl phenol (thymol).

**Figure 1:**
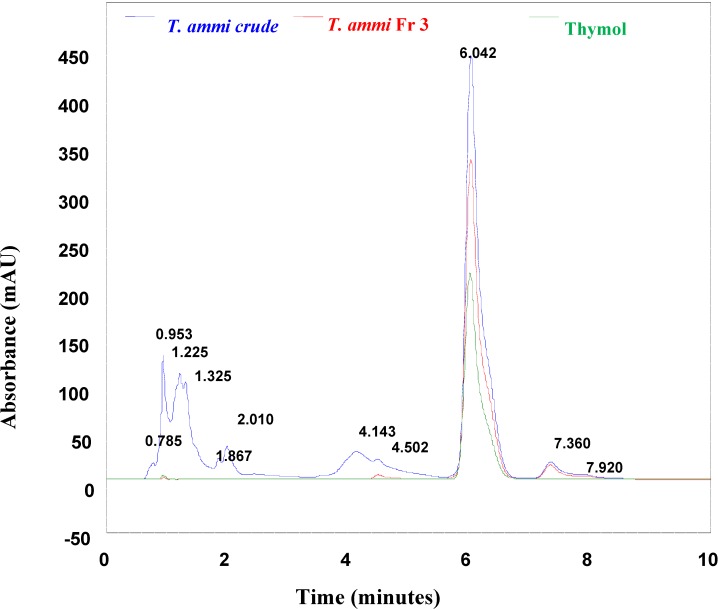
Overlayed HPLC analyses of *T.ammi* extract, active fraction and thymol.

The comparative HPLC analysis of the crude extract, purified fractions and authentic 2-isopropyl-5-methyl phenol (thymol) confirmed that the chromatogram of the active fraction matches that of thymol, with a retention time of 6.04 minutes (Figure 1). Phenolic compounds of natural origin have the desirable property of being soluble in polar solvents. This leads to the possibility of using reverse phase HPLC (RP-HPLC) in their analysis. Sufficient retention time could be achieved by using acidic conditions in order to avoid the presence of ionized forms of the analytes. The combination of RP-HPLC and UV detection is widely used in both qualitative and quantitative analysis of Nature-derived samples containing phenolics. The UV wavelength of 280 nm has proved to be suitable for universal detection of all phenolics [23,24]. Here the thymol content was analyzed using Ultracarb C8 Column using a mobile phase combination of methanol, water and acetic acid at 272 nm and a PDA detector.

### Antifilarial screening

The *in vitro* screening was carried out using adult *S.digitata* worms with the crude extract as well as the fractions collected from the flash columns. Out of ten fractions collected, fractions 3-8 were pooled based on TLC (R_f_ 0.68) carried out with precoated aluminium sheets with the hexane-ethyl acetate (8:1) and these were found to contain the active principle. Other fractions were inactive. The *in vitro* macrofilaricidal activity was assessed by worm motility and MTT reduction assay. Motility assay showed complete inhibition of motility at higher test concentrations tested. Screening was carried out at varying concentrations ranging from 0.001-1.0 mg/mL at two different incubation periods *viz*., 24 and 48 hrs to find out the dose response relationship as well as the effect of duration of exposure to the test material on the viability of the filarial worms. The results of MTT reduction assay are given in Table 1.

*T. ammi* crude extract exhibited macrofilaricidal activity which was quantitatively measured in terms of percentage reduction in formazan formation with respect to the untreated control worms in MTT reduction assay. The corresponding IC_50_ values were 0.067 and 0.019 mg/mL at two different incubation periods 24 and 48 hr respectively. The IC_50_ values for the isolated active principle 2-isopropyl-5-methyl phenol at two incubation periods 24 and 48 hr were 0.024 and 0.002 mg/mL, respectively. The position isomer of the active principle 5-isopropyl-2-methyl phenol also showed macrofilaricidal activity with IC_50_ values of 0.025 and 0.004 mg/mL, respectively, for 24 and 48hr incubation periods. The results showed a dose dependent effect on inhibition of formazan formation by the test materials. It was also observed that at longer exposure period the compounds were more effective showing that the compounds may be slow acting. The worm motility assay and MTT reduction assay have confirmed the macrofilaricidal potential of the fruit extract of *T. ammi* and the fraction obtained after chromatographic purification.

**Table 1:** *In vitro* macrofilaricidal activity of extract of *T. ammi* fruits against *S. digitata* adult females by the MTT reduction assay, in comparison with the active principle and its isomer.

S. No.	Test extract/Compound	Incubation period (hr)	Test concentration (mg/mL)	% Formazan formation inhibition ± s.e (n=12)	IC_50_ (mg/mL)
1	*T. ammi* extract	24	0.01	11.88 + 1.57	0.067
			0.05	30.84 + 1.49	
			0.10	53.59 + 0.96	
			0.50	92.30 + 0.786	
		48	0.005	21.26 + 0.82	0.019
			0.01	36.74 + 1.04	
			0.05	65.12 + 0.48	
			0.10	80.96 + 1.21	
			0.50	93.43 + 0.75	
2		24	0.005	20.77 + 0.93	0.024
	2-isopropyl-5-methyl phenol		0.01	34.29 + 1.16	
			0.05	60.80 + 1.87	
			0.10	76.94 + 0.47	
			0.50	97.61 + 0.61	
		48	0.001	35.43 + 0.93	0.002
			0.005	59.35 + 1.41	
			0.01	71.64 + 0.84	
			0.05	85.39 + 0.45	
			0.10	94.18 + 0.19	
3		24	0.005	24.25 + 0.75	0.025
	5-isopropyl-2-methyl phenol		0.01	31.08 + 2.49	
			0.05	54.00 + 1.15	
			0.10	76.47 + 0.77	
			0.50	95.61 + 0.17	
		48	0.005	25.88 + 1.30	0.004
			0.01	68.82 + 1.31	
			0.05	80.44 + 0.54	
			0.10	95.83 + 0.46	

All the compounds were screened at concentrations ranging from 0.001-1.0 mg/mL. The concentrations showing <1% as well as >99% inhibition are not included in the table.

**Figure 2:**
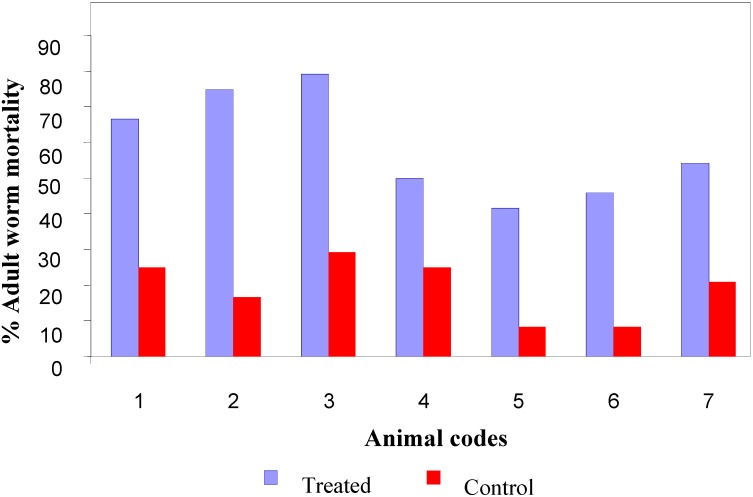
Adult worm mortality in treated and control animals.

The *in vivo* effect of the active principle 2-isopropyl-5-methyl phenol was evaluated against the *B. malayi* parasite in a *Mastomys coucha* model. The observed adult worm mortality in treated and control animals is shown in Figure 2. The mean percentage mortality of adults (58.93%) in the group treated with 50 mg/Kg was significantly (Chi-square=56.2; P<0.0001) higher than that was obtained in the control group (19.05%).

The percent sterilized (48.6%) in the group treated with 50 mg/Kg was significantly (Chi-square=11.54; P<0.0001) higher than that was obtained in the control group (17.8%, Table 2). The results showed that the active principle isolated from *T. ammi* has promising *in vivo* antifilarial activity in terms of adult worm mortality as well as female worm sterility. However the circulating microfilariae were not affected by this compound at a dose level of 50 mg/kg, i.p. x 5days (Table 3).

**Table 2:** Sterilizing effect shown by 2-isopropyl-5-methylphenol in recovered adult worms from treated *Mastomys*.

Group	Dose (mg/Kg) i.p x 5days	Number of recovered females teased	Number of worms sterilized (%)	95% confidence limits
Treated	50	37	18 (48.6)	32.5-64.8
Control	-	73	13 (17.8)	9.0-26.6

**Table 3:** Effect of 2-isopropyl-5-methylphenol on *B. malayi* microfilaraemia density per 10 µL of tail blood in treated and control animals on pre- (0th day) and post-treatment days.

Days	Treated Group (50 mg/Kg.) mean ± S.D	Control Group mean ± S.D
0	29.1 + 13.6	54.3 + 18.8
8	45.7 + 12.0	56.4 + 12.6
15	61.4 + 19.4	61.4 + 18.9
30	73.4 + 23.6	65.9 + 19.6
45	78.0 + 24.9	75.0 + 25.3
60	86.9 + 29.8	84.4 + 20.3
75	109.0 + 31.7	103.9 + 32.8
90	119.6 + 33.6	108.3 + 33.1

Parasitic diseases are responsible for considerable morbidity and mortality throughout the world especially in the tropical and subtropical regions. The morbidity caused by the important tropical diseases, lymphatic filariasis and onchocerciasis, caused by infections with filarial nematodes, are estimated at 951,000 DALYs (Disability-Adjusted Life Year- One DALY represents a lost year of healthy life and is used to estimate the gap between the current health of a population and an ideal situation where everyone in that population would live into old age in full health) for river blindness and 5.5 million DALYs for lymphatic filariasis [25]. Present treatment regimens for these diseases have limitations, as the currently used antifilarial drugs are mainly microfilaricidal, with little effect on the adult worms and so new drugs are urgently required. In this regard, natural products have made and continue to make important contributions to this therapeutic area. This study demonstrates that the crude and purified extracts of *T. ammi* are effective in killing the adult bovine filarial worms *in vitro*. The most important finding in this study is that the active principle has showed promising *in vivo* macrofilaricidal activity as well as female worm sterility against human filarial worm *B. malayi* in *M. coucha*.

Among the most promising advances in the field of drug development is discovering new molecules or novel uses of the already available compounds with known safety and without any side effects. Thymol is a naturally occurring phenolic monoterpene, known for its anti-oxidant and anti-inflammatory potential in various disease conditions [26,27]. Use of thymol-containing preparations for treating various diseases ranging from mouth washes and enteric disorders to skin infections is common [22,28,29]. The antimicrobial effect of (–)-menthol, thymol and linalyl acetate may be due, at least partially, to a perturbation of the lipid fraction of bacterial plasma membranes, resulting in alterations of membrane permeability and in leakage of intracellular materials [30]. Besides being related to physicochemical characteristics of the drugs (such as lipophilicity and water solubility), this effect appears to be dependent on the lipid composition and net surface charge of the bacterial membranes. Furthermore, the drugs might cross the cell membranes, penetrating the interior of the cell and interacting with intracellular sites critical for antibacterial activity.

The essential oils of oregano (*Origanum vulgare* L.) and thyme (*Thymus vulgaris* L.) are effective against *Trypanosoma cruzi*, with higher activity oberved for thyme, and thymol may be the main component responsible for the trypanocidal activity [31]. Thymol showed considerable molluscicidal effect against *Biomphalaria alexandrina*, *Bulinus truncatus* and *Lymnneae natalensis* [32]. Promising antileishmanial and antimalarial potential has also been reported for thymol [33]. The main constituent, carvacrol, but also its positional isomer, thymol, were tested. Interestingly, both compounds retained the same activities observed for the oil. Furthermore, their trypanocidal activity was even stronger (IC_50_ value for thymol: 114 ng/mL, for carvacrol: 149 ng/mL). Both the oil and its two components are devoid of cytotoxicity on mammalian L6 cells (IC_50_ >50 µg/mL). Recently the *in vitro* and *in vivo* antileishmanial and cytotoxic activities of thymol and structural derivatives were compared to those of glucantime [34]. The reported results suggest that thymol and hemisynthetic derivatives have promising antileishmanial potential and could be considered as new lead structures in the search for novel antileishmanial drugs. None of the compounds seems to be toxic to the animals based on corporal weight, behavior, and serum levels of bilirubin, uric acid, and glucose. While complete cures did not occur, the absence of toxicity of these products will facilitate daily oral treatment for longer periods.

Recently preliminary screening of *T. ammi* seed extracts for anthelmintic activity in sheep has been reported [17], but the antifilarial potential of this important medicinal plant has not yet been exploited fully. In the absence of an effective and safe antifilarial agent that could kill the long lived adult filarial worm, it is worth exploring the possibility developing suitable pharmaceutical formulations for the treatment of filariasis and the active principle could be considered as lead structure for development of novel macrofilaricidal agents.

## Conclusions

In summary, the methanolic extract of *T. ammi* has exhibited promising macrofilaricidal activity against adult bovine filarial worm *S. digitata* and the active principle responsible for this has been identified as thymol. The position isomer of thymol namely carvacrol also showed *in vitro* antifilarial activity but to a lesser extent than thymol. The active molecule isolated from *T. ammi* has promising *in vivo* antifilarial activity against human filarial worm *B. malayi* in terms of adult worm mortality as well as female worm sterility.

## Experimental

### General

HPLC grade methanol, glacial acetic acid, analytical grade hexane, chloroform and absolute ethanol (s. d. fine-chem., Mumbai, India), Dulbecco’s Modified Eagle’s Medium (DMEM) and foetal calf serum (Sigma Chemical Co., St. Louis, USA), Strepto-penicillin (Sarabhai Chemicals, Baroda, India) and MTT (3-[4,5-dimethylthiazol-2-yl]-2,5-diphenyl tetrazolium bromide) (Sisco Research Laboratory, Mumbai, India) were used. Millipore filtered water was obtained by passing distilled water through a Milli Q system (Millipore Corp., Milfed, MA). Dried *T. ammi* fruits were purchased locally, identified by a botanist and voucher specimen was kept for future studies. The FT-IR spectrum was recorded on a Shimadzu FT-IR model 8300 (Shimadzu Corporation, Kyoto, Japan). ^1^H-NMR Spectrum was taken on a GSX-400 (JEOL, Tokyo, Japan). Mass spectrum recorded on a GC-MS (Finnigan Mat, Sanjose, CA). Spectrophotometric readings were taken in a SpectraMax Plus (Molecular devices, Sunnyveil, CA) with SoftmaxPro software. HPLC system (ThermoFinnigan, CA) composed of Spectra System P4000 solvent delivery system, Spectra System AS3000 autosampler and Photodiode array (PDA) detector SN4000 was utilized. The output signals were monitored with ChromQuest 4.0 Chromatography workstation.

### Extraction, lead isolation and structure elucidation

Dried fruits of *T. ammi* (about 100 g) were powdered and used for each extraction with analytical reagent grade methanol (500 mL) in a Soxhlet apparatus. After extraction, the solvent was flash evaporated in a rotary vacuum evaporator for concentrating the extract (14.2% residue). A portion of the residue was weighed and reconstituted in ethyl alcohol (30% w/v). This crude extract was screened for macrofilaricidal activity after proper dilutions. Another portion was subjected to bioassay-guided fractionation by flash chromatography. One gram of residue was subjected to SGCC with hexane-ethyl acetate (8:1) as eluent on a 10 g FlashPack SIL connected to a Flashmaster Personal flash chromatography system (Argonaut Technologies Ltd., Mid Glamorgan, UK). Ten fractions (10 mL each) were collected. Fractions 3-5 and 6-8 were pooled based on TLC (R_f_ 0.68) carried out with precoated (silica gel 60F_254_) aluminium sheets (Merck, Germany) with the hexane ethylacetate (8:1) and solvent was removed to give a residue (0.407 g) which melted at 50-51^o^C. IR: 3442, 2961 and 1619 cm^‑1^, characteristic of phenolic monoterpene; ^1^H-NMR: 1.21 (d, 6H), 2.23 (s, 3H), 3.2 (m, 1H), 4.3 (s. 1H), 6.5 (s, 1H), 6.76 (d, 1H), 7.1 (d, 1H). MS: 150 (M^+^), 135, 115, 101, 91, 71 and 57.

### HPLC analysis of T. ammi extract

A simple and sensitive isocratic high performance liquid chromatographic (HPLC) method [35] was used for the determination of active ingredient content in *T. ammi* extract. A 5 μm C_8_ reversed phase Phenomenex Ultracarb analytical column (150 x 4.6 mm) and a mobile phase combination of methanol-water-acetic acid (60:40:2) at the flow rate of 1.5 mL/min at 50^o^C were used for the analysis. The residues from crude extract of *T. ammi* and the fractions obtained from SGCC were dissolved in methanol and analyzed by HPLC using PDA detector seat at 272 nm.

### In vitro screening for macrofilaricidal activity against S. digitata

Adults of the cattle filarial parasite of *Setaria digitata* (Nematoda: Filariodea), were used as the test organism for screening the macrofilaricidal activity. Adult *S. digitata* worms collected from the peritoneal cavity of freshly slaughtered cattle were washed with normal saline (0.85%) to free them of any extraneous material and transferred to DMEM containing 0.01% Strepto-penicillin and supplemented with 10% heat- inactivated foetal calf serum were used for the experiment within an hour as reported earlier [36,37].

*Worm motility assay:* Dilutions of the crude extract (30%w/v) of *T. ammi* were made in ethyl alcohol and screening was done at concentrations ranging from 0.001-1.0 mg/mL. A simultaneous control was kept with the vehicle ethyl alcohol alone in the medium. Two adult female *S. digitata* worms were introduced into each Petri dish. Three replicates each were there for both test and control. The worms were incubated at 37°C for 24 and 48 hrs in an incubator. After the incubation period the number of immobilized worms in each petridish was counted. Immediately after counting, the worms were washed twice with fresh medium and transferred to another set of Petri dishes containing fresh medium, without the test solution, to find out whether any of the immotile worms regained motility. If the worms did not revive, the condition was considered as irreversible and the concentration lethal. Each experiment was repeated twice.

*MTT- formazan colorimetric assay for viability of worms:* *T. ammi* extract was further screened for viability of adult *S. digitata* through a MTT reduction assay following the method reported earlier [38]. Adult female worms were used for this assay. After the exposure of the worms to various concentrations of *T. ammi* (0.001- l.0 mg/mL) in DMEM at 24 and 48 hr incubation period, the parasites were further incubated for 30 minutes individually in phosphate buffered saline (pH 7.4, 0.5 mL) containing MTT (0.25 mg/mL). A control was set up with adult females not treated with the test solution but exposed to alcohol solvent as described in the above experiment. All the test concentrations and controls were in 12 replicates. At the end of the MTT incubation, the worms were transferred to a microtitre plate containing 400 µL of spectroscopic grade dimethyl sulphoxide (DMSO) and allowed to be at room temperature for 1h, with occasional gentle shaking to extract the colour developed. The absorbance of the resulting formazan solution was then determined at 492 nm in a microplate spectrophotometer relative to DMSO blank. As the values of absorption correlate with formazan formation, viability of the worms was estimated as percentage inhibition in formazan formation relative to control worms. Similarly the pooled fractions obtained from flash chromatography also were screened for macrofilaricidal activity. The antifilarial activity of the active principle thymol (2-isopropyl-5-methyl phenol) was compared with the positional isomer of the active compound carvacrol (5-isopropyl-2-methyl phenol).

### In vivo screening for macrofilaricidal activity against B. malayi

The subperiodic strain of *B. malayi* was maintained in a rodent host, *M. coucha*, as reported previously [39]. Male mastomys 6 weeks of age were infected by subcutaneous inoculation of 100 infective larvae (L3) of B. malayi recovered from mosquito vector Aedes aegypti on day 9±1 of infected blood meal in the back region. *M. coucha* with a 5-6-month-old infection, showing a progressive rise in microfilaraemia, were selected for *in vivo* evaluation. A total of 14 infected animals were included in the study. Of these, seven animals were given the active compound at 50 mg/kg intraperitoneally daily for five days while the other 7 animals served as controls.

The micro- and macrofilaricidal efficacy of the active fraction was evaluated as described earlier [40]. A total of 10 μL of tail blood was taken from each animal and spread as a thick smear which was air dried. The smears were dehaemoglobinized with water and later stained with Leishman’s stain. The slides were viewed under a microscope at 10× magnification to assess the microfilarial density in the blood. Blood was taken on day 0 just before the start of treatment, and on days 8, 15, 30, 45, 60, 75 and until day 90 after treatment. Microfilaricidal efficacy in an individual animal was expressed as the percent reduction in the density of microfilariae over the pretreatment level. A compound or fraction was considered microfilaricidal on the basis of a fall in the microfilarial level on day 8 after the treatment. Assessment of the macrofilaricidal/adulticidal efficacy of the extract was carried out by evaluating the percent reduction in adult worm recovery in the treated group compared to untreated animals. Treated and untreated animals were killed on day 90 after the start of treatment, and the heart, lungs and testes were taken out and teased carefully to recover the adult parasites of both sexes. These were examined for their motility, cell adherence on their surfaces, death or encapsulation. All surviving females were teased individually in a drop of saline and the condition of the embryonic stages in the uteri was examined microscopically. Any abnormality or death/distortion detected in the uterine contents, including oocytes, eggs and microfilariae was considered as a sterilization effect of the compound on the female [40].
